# Ultrasensitive *c*DNA Detection of Dengue Virus RNA Using Electrochemical Nanoporous Membrane-Based Biosensor

**DOI:** 10.1371/journal.pone.0042346

**Published:** 2012-08-23

**Authors:** Varun Rai, Hapuarachchige C. Hapuarachchi, Lee Ching Ng, Siew Hwa Soh, Yee Sin Leo, Chee-Seng Toh

**Affiliations:** 1 Division of Chemistry and Biological Chemistry, School of Physical and Mathematical Sciences, Nanyang Technological University, Singapore, Singapore; 2 Environmental Health Institute, National Environmental Agency, Singapore, Singapore; 3 Communicable Disease Centre, Tan Tock Seng Hospital, Singapore, Singapore; Northeastern University, United States of America

## Abstract

A nanoporous alumina membrane-based ultrasensitive DNA biosensor is constructed using 5′-aminated DNA probes immobilized onto the alumina channel walls. Alumina nanoporous membrane-like structure is carved over platinum wire electrode of 76 µm diameter dimension by electrochemical anodization. The hybridization of complementary target DNA with probe DNA molecules attached inside the pores influences the pore size and ionic conductivity. The biosensor demonstrates linear range over 6 order of magnitude with ultrasensitive detection limit of 9.55×10^−12^ M for the quantification of ss-31 mer DNA sequence. Its applicability is challenged against real time cDNA PCR sample of dengue virus serotype1 derived from asymmetric PCR. Excellent specificity down to one nucleotide mismatch in target DNA sample of DENV3 is also demonstrated.

## Introduction

Dengue virus (DENV), a single-stranded RNA positive-strand mosquito-borne virus is of the genus flavivirus which includes the West Nile virus, Tick-borne Encephalitis Virus, Yellow Fever Virus, and several other viruses which may cause encephalitis. DENV is highly infectious and widespread in tropical and subtropical region with epidemic challenge [1]. There are four antigenically different serotypes of the virus (DENV1-4). Dengue virus infection induces long-life protection against the infecting serotype, but it gives only a short time cross protective immunity against the other types. The first infection causes mostly minor disease, but secondary infections has been reported to cause more severe forms of the disease: Dengue Hemorrhagic Fever (DHF) and Dengue Shock Syndrome (DSS). To contain the effect of epidemic spread of dengue infection, its early detection is deemed necessary with follow-up vector control measures and a responsive medical support system. Reverse-transcription polymerase chain-reaction (RT-PCR) and monoclonal antibody capture-enzyme linked immunosorbant assay (MAC-ELISA) are the current laboratory tests used worldwide to diagnose dengue infections [2]. However PCR based methods require tedious quality control and meticulous handling of biological sample to reduce chances of observing false positive results due to unwanted amplification of contaminants. Conversely, non-structural dengue proteins, NS1 and specific immunoglobulin G (IgG) or M (IgM) dengue antibodies, have been frequently preferred as the biomarkers for dengue detection in serological tests which require less elaborate sample preparation procedure but are of poorer specificity and sensitivity in comparison with PCR methods [2].

Nanoporous membrane based biosensors have been used to detect small molecules proteins, cells, virus, metal ions and DNA [3]–[8]. Moretti et al. used silicon nitride based nanopore biosensor to detect DNA while monitoring ionic current through pore [9]. Wang and co-workers have used chitosan-carbon nanotubes as matrix to attach probe DNA and monitor hybridization into electrochemical and fluorescence signal [10]. Ding et al. used aptamer coated glass nanopore biosenosor to detect protein immunoglobulin [11]. Recently, interesting solid state bio-functionalized nanopores biosensors to detect complementary target molecules present in solution and using electrophoretically drawn movement through the nanometric channel has been reported by Mussi et al. [7], [12]. PNA modified synthetic ion channels have been used in nanoconfined environment for target analyte hybridization and sensing [13]. Notably, gold and alumina nanotubule membranes have been extensively applied to detect bioanalytes [14]. These nanopores and tublules are functionalized with biomolecular recognition element and binding of target analyte with molecular recognition element selectively blocks the nanopore leading to decrease in ionic current.

Herein we use nanoscale porous alumina-coated electrode for developing sensing surface to detect specific sequence of the DENV genome. The unique property of high aspect ratio and high surface area of porous nanoelectrodic surface is exploited to increase probe loading and extend the limit of detection of target analyte in electrochemical analysis [15]–[17]. Electrochemical anodization of aluminium results into a nano porous multi-channel alumina structure with pore size range from 10 to 150 nm and density of about 1×10^10^ pores cm^−2^ [18]. This anodization technique is comparatively easier than conventional lithographic methods. We attach probe DNA molecules covalently in the alumina nano channels which selectively bind to 31 mer specific DENV DNA target sequence. Binding of target complementary DNA to probe inside nanochannels causes changes in mass transfer of redox species Fe(CN)_6_^4−^ through it due to blocking of the pores. Mass transfer changes through alumina nanopores are translated into electrochemical signal using differential pulse voltammetric technique (DPV). DPV oxidative peak current of Fe(CN)_6_^4−^ successively drops with increase in target complementary DNA concentration. This nanoporous alumina based DNA biosensor is easy to prepare and shows low detection limit ∼10^−12^ M with wide linearity range of 6 orders which are similar to other amplified electrochemical biosensors. Apart from low detection limit, the nanoporous alumina based DNA DENV biosensor selectively differentiates one base mismatch in target DENV3 DNA sequence therefore differentiating DENV1 from DENV3.

## Materials and Methods

### Reagents

DNA probe sequence of DENV 1 attached on electrode (5′NH_2_(CH_2_)_6_ GCGGTAAC CTC TGA TGA ACA ACC AAC GGA AAA AGA CGG G GTTACCGC - 3′), target analyte cDNA of DENV 1 complementary to the probe (3′- GAG ACT ACT TGT TGG TTG CCT TTT TCT GCC C -5′), and cDNA of DENV 3 single nucleotide mismatch (3′-GAG ACT ACT TGT TGG TTG CCT TCT TCT GCC C -5′), potassium hexacyanoferrate (II) trihydrate, potassium hexacyanoferrate (III), chromic acid, phosphoric acid (85%), 3-aminopropyltrimethoxysilane (APS), glutaraldehyde (25wt% solution in water), propylamine, sodium chloride, platinum wire 99.99% (76 µm diameter), 1.0 M tris (2-carboxy-ethyl) phosphine hydrochloride (TRIS buffer) of pH 7.0, were obtained from Sigma Aldrich. All target analyte DNA solutions were prepared using 1.0 M TRIS buffer pH 7.0. 1 M and 1× phosphate buffer saline (PBS) solution (pH 7.2) was obtained from 1st Base. Alumina powder (1 µm and 0.3 µm) were purchased from Allied High Tech Products, Inc. epoxy structural adhesive DP 760 was obtained from 3 M Technologies (S) Pte Ltd. Alumina target 99.99% purity was obtained from Optoelectron Technologies. All reagents were used as received, unless otherwise stated.

### Procedure for analyses of DNA targets

The nanoporous alumina based DNA biosensor was thermostated in the complementary target (DENV1) solution at 45°C and target with one base mismatch (DENV3) at 53°C respectively for 30 min followed by cooling up to room temperature to allow complete hybridization. The biosensor was subsequently rinsed with ultrapure water to remove any unhybridized target, followed by electrochemical measurements at room temperature. Electrochemical measurements were performed using CV and DPV techniques, DPV signal was recorded of bare alumina electrode followed by its successive modification with probe DNA and aftermath hybridization with complementary target to investigate electrochemical response of the biosensor. In order to test the applicability of the Nanoporous alumina membrane based biosensor in real sample analysis, it was challenged with complementary DNA (cDNA) PCR amplicons of Dengue virus 1 (DENV1) isolated from human serum. A 31 bp region between nucleotide positions 90 and 120 of DENV1 genome (NCBI Ref Seq NC_001477.1) was selected as the target sequence on the probe of the biosensor (Table 1). DENV1 RNA was extracted from a virus suspension using the QIAmp viral RNA Mini Kit (Qiagen, Hilden, Germany) according to manufacturer's instructions. DENV cDNA was prepared from the extracted RNA using the Superscript™ III first-strand synthesis system (Invitrogen Corporation, Carlsbad, USA) according to manufacturer's guidelines. PCR amplification was performed in a Veriti® Dx 96-well thermal cycler (Applied Bio systems, Singapore) using primers that flanked the DENV1 genomic region from 19 to 201 nucleotides. An asymmetric PCR protocol (Forward: Reverse primer ratio = 1∶10) was used to generate an excess of the reverse strand which is complementary to the probe sequence. The amplification was performed using the Phusion® Flash high-fidelity PCR master mix (Thermo Fisher Scientific Company, Espoo, Finland) as recommended by the manufacturer. The reaction utilized 2 µl of cDNA as the template in a protocol as follows; an initial denaturation at 98°C for 30 sec followed by 35 cycles of 98°C for 5 sec, 67°C for 8 sec, and 72°C for 10 sec with a final extension of 72°C for 1 min. PCR products were visualized by 1.5% agarose gel electrophoresis Fig. 4 (A). In order to confirm that the amplified products flanked the region encoding the probe, several amplicons were sequenced at a commercial lab using Big Dye Terminator Cycle Sequencing kit, according to manufacturer's instructions (Applied Biosystems, Foster City, CA, USA). PCR products were subsequently diluted by 10 fold up to four serial dilutions for detection by the biosensor.

**Table 1:** Sequences of the 183 bp target, two primers and the probe.

DENV I DNA Probe	5′- CTCTGATGAACAACCAACGGAAAAAGACGGG - 3′
Forward primer :	5′- ACCGACAAGAACAGTTTCAAATCG - 3′
Reverse primer :	5′- CCTTTTGAGAATCTCTTCGCCAAC -3′
Target analyte (183 bp)	3′-TGGCTGTTCTTGTCAAAGTTTAGCCTTCGAACGAATTGCATCAAGATTGTCAAAAAATAATCTCTCGTCTAGAGACTACTTGTTGGTTGCTTTTTTCTGCCGAGCTGGCAGAAAGTTATACGACTTTGCGCGCTCTTTGGCGCACAGTTGACAAAGTGTCAACCGCTTCTCTAAGAGTTTTCC-5′

### Fabrication of nanoporous membrane-based DNA biosensor

**Figure 1:**
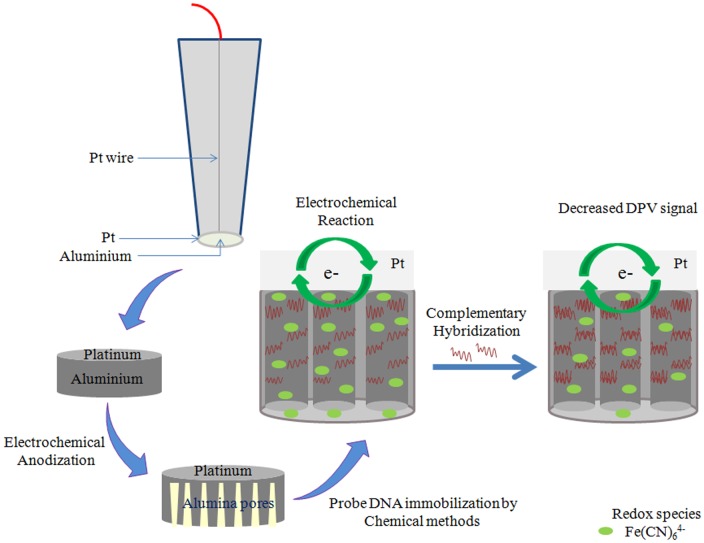
Schematic. Scheme of construction and operation for nanoporous alumina membrane based DNA biosensor.

The fabrication design and operating principle of alumina membrane based DNA biosensor are shown in Fig. 1. Homemade electrodes were fabricated using chemical resistant epoxy resin (RS Components Pte Ltd), micropipette tips and 99.99% platinum wire (76 µm diameter, Sigma Aldrich). The platinum wire was aligned in the center of the micropipette tips and sealed within epoxy resin. The platinum wire was subsequently soldered to a copper wire and the connection was sealed with epoxy resin. The fabricated platinum wire electrodes were polished with 1.0 µm and 0.3 µm diameter alumina slurry and sonicated in ultrapure water (with resistivity of more than 18 Ω). Sub-micrometer thick aluminum films were sputter coated over the platinum electrodes using 99.999% purity aluminum target, Denton discovery® 18 Sputtering System and sputtering power of 100W in an atmosphere of research-grade Ar at 5×10^−3^ Torr. Anodization of aluminum coated electrodes was conducted using a previously described method of surface contact anodization [18].

5′-aminated DNA probes was covalently attached onto nanoporous alumina using glutaraldyhyde cross linking [19]. The nanoporous alumina electrodes were immersed in 5% APS solution for an hour and dried in vacuum oven for 30 minutes at 45°C after thorough washing with acetone and drying with argon. APS activated naonoporous alumina electrodes were immersed in glutaraldehyde for 12 hours, followed by thorough washing with ultrapure water and drying with argon. ∼50 µL of 100 µM of 5′ aminated probe DNA probe solution was added onto the surface and kept at high humidity overnight. The electrodes were subsequently rinsed with 1 M NaCl to remove any non-specific adsorbed DNA and dried in argon. Few drops of 10^−6^ M of propylamine was added on to the nano porous alumina electrodes and left for 6 h. to neutralise excess glutarldehyde and facilitating efficient hybridization with complementary target during thermostatic incubation. Followed by, thorough washing of the electrodes was performed using ultrapure water and then dried in argon.

### Electrochemical measurements

Electrochemical behaviours of the alumina modified electrodes were investigated using cyclic voltammetry and differential pulsed voltammetry (DPV) techniques (CHI 750 potentiostat/galvanostat, data acquisition software) in the presence of 1.0 mM Fe(CN)_6_^4−^ in 1 M 1× phosphate buffer solution, pH 6.8 using three electrode system. The nanoporous alumina pipette electrode biosensor was used as working electrode and all potentials and currents were measured with respect to the Ag/AgCl (1.0 M KCl)) reference electrode and Pt gauze counter electrode. Differential pulse voltammetry was carried out using 50 ms pulse width, 50 mV pulse height, pulse period of 200 ms and potential increment of 1 mV and CV was recorded in potetinal window of −0.1 to 0.7 V with scan rate of 50 mv/sec

## Results and Discussion

### Biosensing mechanism and sensing signals derived from differential pulse voltammetry (DPV)

**Figure 2:**
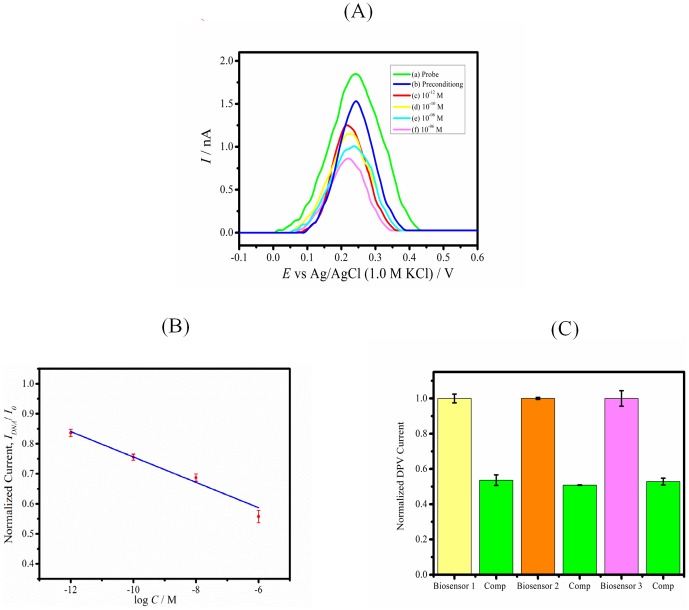
Complementary target detection and reproducibility. (A) Differential pulse voltammetry current signal response of (a) biosensor, (b) preconditioning of biosensor and towards increasing concentration of complementary target: (c) 10^−12^,(d) 10^−10^,(e) 10^−08^ and (f)10^−06^ M. DPV currents were offset to 0 µA to allow comparison of results and all measuring solutions contain 1×, pH 7.2 PBS electrolyte solution. (B) Averaged normalized current signal response best fitted linearly with log C of complementary target. Error bars and points represent average standard deviations derived from single biosensor with three consecutive measurements (C) Normalized DPV current signal response of different biosensors 1, 2 and 3 towards identical complementary analyte at 10^−6^ M concentration. Error bars correspond to standard deviations obtained from 3 consecutive DPV measurements.

As Dengue virus genome is single stranded RNA genome, therefore unique 31 mer ssDNA complementary sequences of DENV1 and DENV3 RNA genome were selected as target analyte for electrochemical detection. In the following, we investigate the mass transfer of redox species Fe(CN)_6_^4−^ inside the nanopores as shown in Figure 1 using the DPV method. Significant changes in DPV signal are observed when the DNA probe is attached within the pores and when complementary targets are bound inside the alumina nanochannels. In addition, Figure 2 (A) shows drop down in the differential oxidative peak current of Fe(CN)_6_^4−^ with increase in target concentration of DNA over wide concentration range. We attribute this to target DNA (∼3–5 nm size) binding to probe DNA chemically immobilized inside the alumina nanochannels minimizes the pore size resulting in the decrease of mass transfer of redox species Fe(CN)_6_^4−^ towards the sensing electrode. It is equally possible that in this complementary binding event, the resulting double helix DNA structure holds more negative charge which repels Fe(CN)_6_^4−^ resulting in less accessibility to the sensing electrode. However, when the experiment is repeated with neutral redox species, ferrocenemethanol, decreasing DPV current signals of the biosensor were observed after incubation with increasing complementary DNA targets (Plot not shown). Thus decrease in DPV peak current is seen as selective binding of target with probe DNA.

### Analytical performance

Figure 2 (B) shows the plot of biosensor current signal responses versus the logarithm of complementary ssDNA target concentration in 1 mM Fe(CN)_6_^4−^ of supporting electrolyte 1× PBS buffer (pH 7.4). Excellent linearity with 6 orders of magnitude from 10^−12^ to 10^−6^ M (R^2^ = 0.98) for DPV current signal response was obtained. Detection limit was determined from the minimum DNA concentration which caused the change in DPV current signal response equivalent to three times the average background noise in the absence of DNA target. Detection limits for a 31-mer DNA sequence of DENV 1 was 9.55×10^−12^ M, present significant improvement of 5–6 orders over DNA sensors based on colorimetry, optical and fluorescence [20]–[22]. Figure 2 (C) shows the plot of normalized DPV current signal response of different biosensors 1, 2 and 3 towards identical complementary analyte (10^−06^ M concentration). Three different biosensors show standard deviation of 3.4% in normalized DPV current signal response towards identical complementary analyte of 10^−6^ M.

### Specific response towards one base-pair mismatch of DENV3 sequence

**Figure 3:**
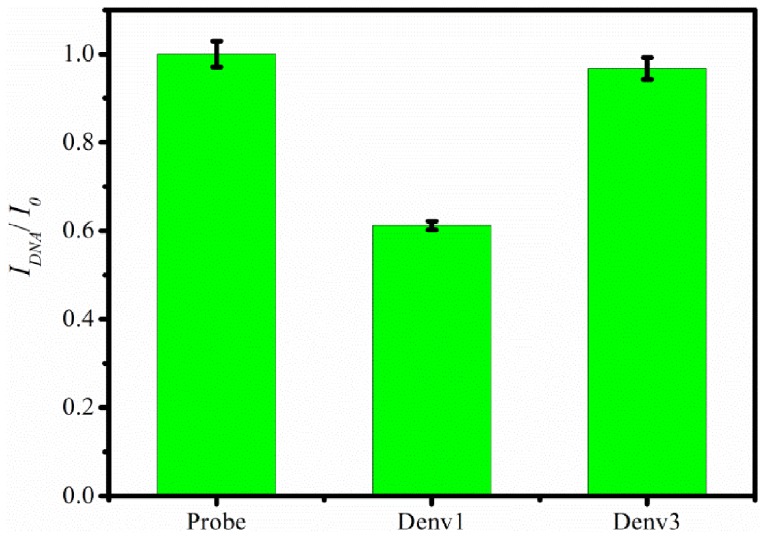
Specificity and one base-pair mismatch DENV3 detection. Changes in normalized differential current signal of the biosensor probe towards 10^−8^ M 31-mer complementary target sequence (DENV 1) and single-base mismatch target sequence (DENV3) respectively. Error bars correspond to standard deviations obtained from 3 consecutive DPV measurements.

Figure 3 shows the normalized current signal response of nanoporous alumina based biosensor towards target containing DNA sequence commonly found in DENV1 with exactly complementary and DENV3 with single-base mismatch in middle position, respectively. These figures of merit including a relatively rapid analysis time of 45 min presents a significant improvement in DNA detection limits over existing non-PCR methods and is comparable to state-of-the-art enzyme based amplified E-DNA sensor, electrochemically amplified DNA sensor and impedimetric DNA sensor [23]–[25].

It is noteworthy to mention that though the current signal error is relatively small (5% error), because of the logarithmic dependence of the concentration range, each measurement gives an error of ca. one order of magnitude in the DNA concentration.

During analysis, the complementary target (DENV1) was thermostated with biosensor at 45°C, which is fairly lower than *T*_m_ 63°C melting temperature calculated using nearest Neighbour thermodynamics based software (biomath Tm Calculators). For the sequence with one mismatch in the middle position, *T*_m_ (53°C) is also lower than the complementary sequence. To achieve selective discrimination of target DNA analyte with one base mismatch, the biosensor was thermostated at 53°C for 30 min. Two complementary strands of DNA will be stacked/hybridized with each other at all temperature lower than its melting temperature above which both strands will be unhybridized and melts away. Melting/hybridization temperature of target analyte DNA of one base mismatch (*T*_m_ 53°C respectively) was suitably exploited in thermostatic incubation to obtain selective discrimination against complementary target. The complementary target (DENV1) and sequence with one nucleotide mismatch (DENV3) can be easily differentiated, which demonstrates this nanoporous alumina membrane based biosensor is fairly selective down to single nucleotide mismatch as shown in Figure 3. Therefore biosensor shows high specificity and selectivity up to one base mismatch in target analyte DNA sequence.

### Regeneration of biosensors with subsequent heating

**Figure 4:**
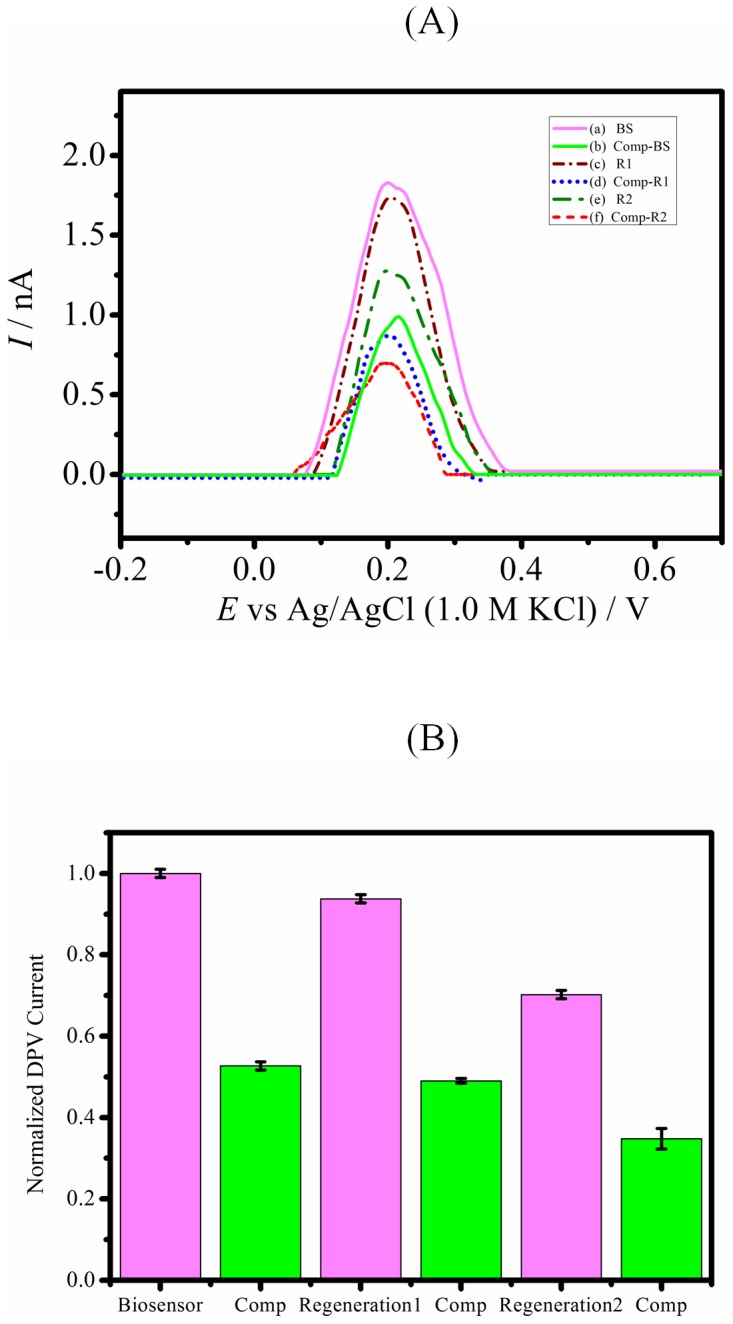
Regeneration of biosensors. (A) Differential pulse voltammetry current signal response of (a) BS, biosensor (b) Comp-BS, biosensor towards complementary analyte 10^−06^ M (c) R1, Regenerated biosensor after first heating cycle (d) Comp-R1,first regenerated biosensor towards complementary analyte 10^−06^ M (e) R2, Regenerated biosensor after second heating cycle. (f) Comp-R2, second regenerated biosensor towards complementary analyte 10^−06^ M. DPV currents were offset to 0 µA to allow comparison of results and all measuring solutions contain 1×, pH 7.2 PBS electrolyte solution. (B) Normalized DPV current signal response of biosensor, first regenerated and second regenerated biosensor towards identical complementary analyte 10^−06^ M. Error bars correspond to standard deviations obtained from 3 consecutive DPV measurements.

Figure 4 shows the DPV current (A) and normalized signal response (B) of one biosensor, its subsequent binding with identical complementary analyte solution 10^−6^ M and its responses after two regeneration cycles. As can be seen that DPV peak current drops when complementary target binds, and in case of heating at 75°C biosensor DPV currents increases significantly up to approximately initial signal response value that is consistent with unbinding of complementary with probe attached into the nanochannels of alumina membrane structure. DPV peak signal response further decreases with binding of complementary with subsequently regenerated biosensor. Subsequently the same biosensor is subjected to second regeneration cycle and its DPV peak current is lower than its first regenerated signal because of possible electrode surface fouling while heating. However ratio of DPV signal response after complementary binding versus biosensor with unhybridized probe remains almost constant at 4.9–5.4. Thus, the used biosensor can be regenerated up to 2–3 cycles with very good reproducible normalized signal response by incubating in a pH 7.0, 0.5 M Tris buffer for 30 min at 75°C. After three regeneration cycles, the porous alumina structure tends to dislodge from the electrode.

### Detection of real time PCR DNA sample derived from DENV1 genomic RNA

**Figure 5:**
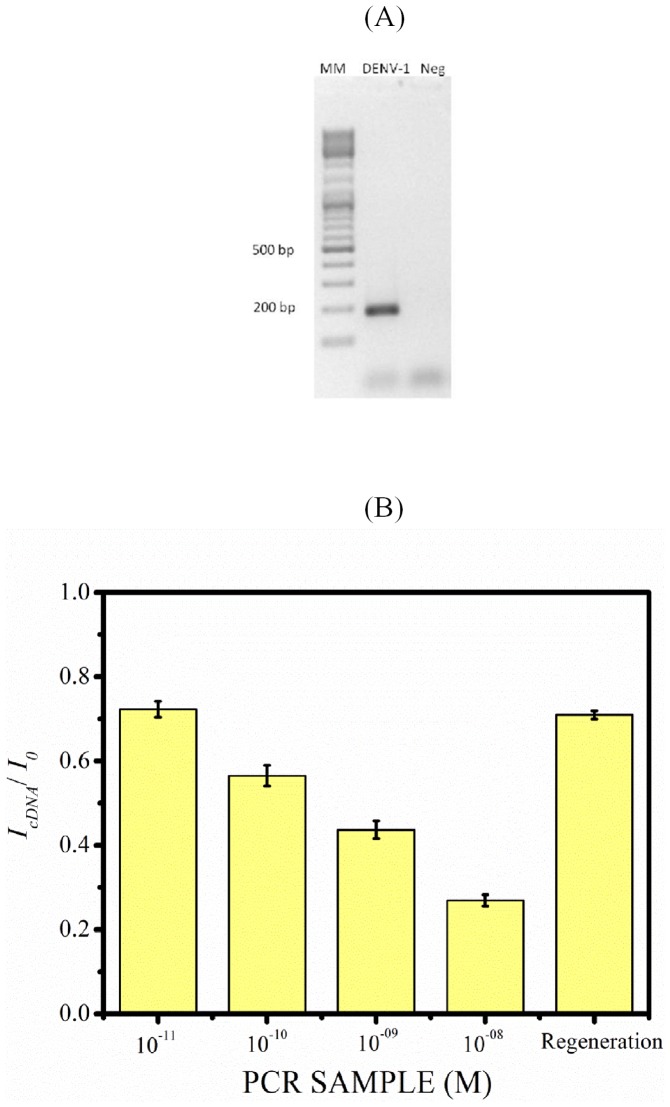
Detection of real time PCR DNA sample derived from DENV1 genomic RNA. (A) Electrophoresis analysis of the 183 bp region of DENVI amplified using asymmetric PCR. (B) Normalized differential current signal response of biosensor towards this real time cDNA PCR sample of 10^−11^, 10^−10^, 10^−9^ and 10^−8^ M, derived from DENV1 genomic sequence using asymmetric PCR. Error bars correspond to standard deviations obtained from 3 consecutive DPV measurements.

Figure 5. (A) Shows gel electrophoresis picture of 183 bp amplicon derived from DENV1 genome using asymmetric PCR method. Figure 5 (B) Shows normalized differential current signal response of nanoporous alumina membrane based biosensor towards real time cDNA PCR sample, derived from DENV1 genomic sequence RNA using asymmetric PCR method. To challenge the performance of the nanoporous alumina based DNA biosensor, it was tested against real time cDNA PCR sample. As can be seen in Figure 5. (B) there is successive dropdown in normalized current signal response of biosensor towards increasing concentration of cDNA PCR samples of DENV1. The biosensor can be regenerated after exposure to the series of diluted PCR amplicon samples using 75°C, 25–30 min heating cycle. This demonstrates the potential use of the method in monitoring pathogens that may be crucial in successful implementation of epidemiologically predicted medical response.

## Conclusions

The biosensor shows excellent performance toward complementary target analyte and genomic DNA derived from PCR with wide linearity and high specificity down to one base mismatch. Its preparation is very simple and relatively easy to carve nanostructure than conventional lithography e.g. electron beam or focussed ion beam. This biosensor exploits only Fe(CN)_6_^4−^ to derive DPV sensing signal in contrast to other amplified sensing mechanism where redox species are labelled/attached in the probe DNA where additional synthetic and purification steps are included.
